# Shunt complications and revisions in children: A retrospective single institution study

**DOI:** 10.1002/brb3.2390

**Published:** 2021-10-17

**Authors:** Nadia Mansoor, Ole Solheim, Oddrun A. Fredriksli, Sasha Gulati

**Affiliations:** ^1^ Department of Neurosurgery St. Olavs University Hospital Trondheim Norway; ^2^ Department of Neuromedicine and Movement Science Norwegian University of Science and Technology (NTNU) Trondheim Norway

**Keywords:** Hydrocephalus, ventriculoperitoneal shunt, ventriculoatrial shunt, cystoperitoneal shunt, shunt complications, shunt revision

## Abstract

**Introduction:**

Shunt surgery in children is associated with high revision and complication rates. We investigated revision rates and postoperative complications to specify current challenges associated with pediatric shunt surgery.

**Methods:**

All patients aged < 18 years admitted to St. Olavs University Hospital, Norway, from January 2008 through December 2017, who underwent primary shunt insertions, were reviewed. Follow‐up ranged from 1 to 10 years. Ventriculoperitoneal, cystoperitoneal, and ventriculoatrial shunts were included. All subsequent shunt revisions and 30‐day postoperative complication rates were registered.

**Results:**

81 patients underwent 206 surgeries in the study period. 47 patients (58%) required minimum one revision during follow‐up. In 14 (29.8%), the first revision was due to the misplacement of hardware. Proximal occlusion was the most common cause of revision (30.4%), followed by misplacement (18.5%) and infection (9.6%). Young age and MMC were associated with revision surgery in a univariable analysis, but were not significant in multivariable analyses. Congenital hydrocephalus was associated with infection (*p *= .028). In approximately 30% of procedures, complications occurred within 30 days postoperatively, the most common being revision surgery. In approximately 5% of the procedures, medical complications occurred.

**Conclusion:**

Children are prone to high revision and complication rates, and in this study, misplacement of hardware and proximal occlusion were the most common. Complication rates should not be limited to revision rates only, as 30‐day complication rates indicate a significant rate of other complications as well. Multi‐targeted approaches, perhaps focusing on measures to reduce misplacement, may be key to reducing revision rates.

## Abstract 2

Children with shunts (ventriculoperitoneal ventriculoatrial) are prone to high revision and complication rates, and in this study, misplacement of hardware and proximal occlusion were the most common. Complication rates should not be limited to revision rates only, as 30‐day complication rates indicate a significant rate of other complications as well. Multi‐targeted approaches, perhaps focusing on measures to reduce misplacement, may be key to reducing revision rates.

## 1 INTRODUCTION

The global prevalence of hydrocephalus (HC) in the pediatric population has been estimated in several studies to be approximately 1 in 1000 births, with medium‐to‐low‐income countries having a significantly higher incidence than high‐income countries (Dewan et al., 2019; Isaacs et al., 2018; Kahle et al., 2016). The most common etiologies in high‐income countries are HC after intraventricular hemorrhage (IVH) in the premature, aqueduct stenosis, myelomeningocele (MMC), and tumors/malignancy, while in low‐income countries, CNS infection is a dominant culprit (Kahle et al., 2016).

Shunting and ventriculostomy are the two permanent treatment methods of CSF diversion. Unfortunately, shunting in the pediatric population is associated with high shunt failure rates/revisions rates ranging from 20% to 85% (Berry et al., 2008; Bir et al., 2016; Gupta et al., 2007; Merkler et al., 2017; Reddy et al., 2014; Riva‐Cambrin et al., 2016; Rossi et al., 2016; Stein & Guo, 2008; Stone et al., 2013; Wu et al., 2007). Complication rates in terms of revision surgery have frequently been examined and the literature is extensive. Predictors such as young age have been suggested as an individual risk factor (Berry et al., 2008; Reddy et al., 2014; Riva‐Cambrin et al., 2016; Tuli et al., 2000), whilst others have found specific etiologies to predict shunt failure (Berry et al., 2008; Reddy et al., 2014; Tuli et al., 2000). Other studies have again failed to establish any specific risk factors (Riva‐Cambrin et al., 2016; Rossi et al., 2016).

In the literature, complications rates are often limited to revision rates as a measure of shunt failure or success. However, in an adult population, we found that complications extend beyond revision surgeries alone, with overall 30‐day complication rates of 45% in a cohort with 20.7% revision rates (Mansoor et al., 2020). We believe that reporting revision rates alone might represent a great underrepresentation of the actual challenges hydrocephalic patients face. Also, failure to assess all aspects of shunt surgery and its complications may limit the ability to improve outcomes by targeting a wider range of complications.

In the current study, we aimed to similarly examine the pediatric population, and in addition to assessing revision rates, we sought to systematically classify the presence of other complications such as postoperative extracranial infections and other medical and surgical complications.

## 2 METHODS

Ethical approval and waiver of the requirement for obtaining patient consent were granted by the Regional Committee for Medical Research, REK 2017/1796.

### 2.1 Data collection

In the current study, we retrospectively reviewed all children ages < 18 years admitted to St. Olavs University Hospital from January 1, 2008 to December 31, 2017. Follow‐up was 1–10 years. Our neurosurgical department serves the well‐defined catchment region of Central Norway, and receives referrals from the seven hospitals in the region. All patients who underwent primary shunt surgery within the given time period were included. Shunt insertion included ventriculoperitoneal (VP), ventriculoatrial (VA), cystoperitoneal (CP), and cystoatrial (CA) shunts. Lumboperitoneal shunts were not included. Subsequent need for shunt revisions were recorded for the period from January 1, 2008 to December 31, 2018; all patients were therefore followed for a minimum of 1 year. We used the Nomesco Classification of Surgical Procedures to identify patients who were eligible for inclusion with the codes AAF 00, AAF 05, AAF 15, AAF 20, AAF 25, AAF 40, AAF99, JAL 50, and JAL 51. All patients were then reviewed individually by one author and included if primary shunt surgery was performed during the given time period.

Data collection included the patients’ age at primary shunt insertion, indication for shunt insertion, type of valve, type of shunt, number of revisions, and causes of revision surgery. In terms of indication or etiology, we classified children as having congenital HC when HC was discovered on prenatal examination (on ultrasound or MRI), present at birth or pathology discovered at or closely after birth resulting in HC. This included patients with Dandy–Walker malformation, Blakes pouch, and so forth, as well as aqueductal stenosis, innate syndromes, and all non‐acquired causes. Arachnoid cysts, although considered congenital, was also given its own entity, as the need for CSF diversion is generally varied and not necessarily a condition diagnosed in infancy. MMC, being a generally rare condition often with multiorgan pathology, was also given its own entity. Chiari I malformation, although perhaps congenital, was given its own entity as many do not require treatment, and shunt dependency usually does not manifest in infancy/early childhood. IVH was given its own entity if hemorrhage was verified by imaging (ultrasound or MRI), being an acquired condition associated with prematurity. Patients were categorized as infantile HC when no obvious congenital pathology was found (i.e., prenatal ultrasound, postnatal UL, or MRI), but hydrocephalus became apparent within the first year of life.

We registered the occurrence of surgical and medical complications for all procedures (primary shunt insertion and all revision surgeries) according to the classification system for neurosurgical complications defined by Ibañez et al. (2011). The highest grade of complication was noted for each procedure.

### 2.2 Data analysis

All data were analyzed using SPSS, IBM, version 25. Categorical variables were presented as frequencies with percentages. Continuous variables were presented with mean, medians, range, and standard deviation. Continuous variables were tested for normality using Shapiro–Wilk test and visualized using Q–Q plots. The chi‐squared test and the independent *t*‐test were used to compare the patient group who underwent revision surgery during follow‐up with the patient group that did not require shunt revision for categorical and continuous data, respectively. In cases of skewed data, the Mann‐Whitney U test was used compare the two groups. To examine possible predictors for the need of revision surgery, we developed a cox regression model. Age, gender, and the different etiologies were entered into a univariable cox regression model. The most common etiologies, compromising more than 10 patients each, as well as factors that were significant (*p* < .05) in the univariable analysis, were then included in a multivariable analysis. Similarly, potential predictors for the occurrence of infection were assessed. To examine the relationship of the presence of serious surgery‐related complications (grade III or IV) *or* the need for revision surgery, we developed a binary regression model.

## 3 RESULTS

### 3.1 Patient demographics

An overview of patient demographics and univariable comparisons of patients undergoing revision surgery and the non‐revised group are presented in Table 1. A total of 81 patients were included in the study. Median age was 8 months (range 0–207 months) and 54.3% of the included patients were male. 88.9% of all patients were alive at the time of end of follow‐up (31 December, 2018). The most common indication for shunt surgery was congenital hydrocephalus (23.5% of patients). This included children with innate syndromes, cysts (Dandy–Walker malformation, Blakes pouch, etc.), as well as aqueduct stenosis and all HC diagnosed prebirth or assumed present at birth. The second most common cause was tumor/malignancy with 17.3% of patients, followed by arachnoid cysts in 13.6% of cases. The case of “other” was a case of venous sinus thrombosis with secondary intracranial hypertension. More than 5‐year follow‐up data was available for 54 (66.7%) of patients. Median follow‐up time was 73.0 months, range: 0–131 months.

**TABLE 1:** Patient characteristics

	No. of patients	Revision group	Non‐revision group	
	*n* = 81 (%)	*n* = 47 (%)	*n* = 34 (%)	*p*‐value
**Age in months** **^a^**	8.0 (0–207)	3.0 (0–191)	71.5 (0–207)	**<.001**
**Gender: male**	44 (54.3)	27 (61.4)	17 (38.6)	.507
**Alive at end of observation**	72 (88.9)	44 (61.1)	28 (38.9)	.156
**Indication**				
Congenital	19 (23.5)	15 (78.9)	4 (21.1)	***.035***
Tumor/malignancy	14 (17.3)	3 (21.4)	11 (78.6)	***.003***
Arachnoid cyst	11 (13.6)	5 (45.5)	6 (54.5)	.513
Infantile	7 (8.6)	5 (71.4)	2 (28.6)	.693
MMC	7 (8.6)	7 (100)	0 (0)	***.020***
IVH	6 (7.4)	4 (66.7)	2 (33.3)	1.00
ICH	5 (6.2)	3 (60.0)	2 (40.0)	1.00
Chiari I	4 (4.9)	2 (50.0)	2 (50.0)	1.00
Unspecified	3 (3.7)	1 (33.3)	2 (66.7)	.569
Traumatic	2 (2.5)	0 (0)	2 (100)	.173
Meningitis	2 (2.5)	2 (100)	0 (0)	.507
Other	1 (1.2)	0 (0)	1 (100)	.420
**Duration** ^a^	55.0 (25–226)	53.0 (25–120)	57.5 (29–226)	.605
**Prior EVD**	22 (27.2)	12 (54.5)	10 (45.5)	.698
**Frontal drain**	57 (70.4)	29 (50.9)	28 (49.1)	.045
**Type of shunt**				
VP	70 (86.4)	41 (58.6)	29 (41.4)	1.00
CP	11 (13.5)	6 (54.5)	5 (45.5)	1.00
**Type of valve** ^b^				
Medtronic Strata II	55 (67.9)	29 (52.7)	26 (47.3)	.160
Codman Certas Plus	9 (11.1)	4 (44.4)	5 (55.6)	.481
Codman Hakim	3 (3.7)	1 (33.3)	2 (66.7)	.569
Medtronic PS Medical Ultra Small	13 (16.0)	12 (92.3)	1 (7.7)	**.006**

^a^ Expressed as median (range).

^b^ *n* = 81. One patient did not receive a valve.

Comparing the revision group with the non‐revision group, we found that patients with congenital and MMC were more likely to undergo revision surgery (78.9% vs. 21.1% with *p* = .035 and 100% vs. 0% with *p* = .020, respectively). Patients with tumor/malignancy were less likely to undergo shunt revision (78.6% vs. 21.4%, *p* = .003). Comparing the two groups, we found significant difference in age in the revision group versus the non‐revision group, with the revision group being significantly younger (median 3.0 months in the revision groups versus median 71.5 months in the non‐revision group, *p* < .01).

A univariable cox analysis showed that age was inversely associated with the need for shunt revision (*p* = .003, HR 0.88), while the diagnosis of MMC was directly associated with shunt revision (*p* = .008, HR 3.0). Frontal drain was inversely associated with revision (*p* = .041, HR 0.54). Gender and other etiologies were not significant in a univariable analysis. Age was still inversely associated (*p* = .019, HR 0.90) with the need for revision in a multivariable analysis testing for age, gender, and the most common etiologies, compromising 54.3% of all patients (congenital HC, tumor/malignancy, arachnoid cyst). Congenital HC was associated with infection in a univariable analysis (*p* = .028, HR 4.61), and was also significant in a multivariable analysis testing for age, sex, and the most common etiologies, congenital HC, tumor/malignancy, and arachnoid cyst (*p* = .033, OR 6.09).

### 3.2 Shunt surgery characteristics

An overview of shunt surgery characteristics and differences between the revision and non‐revision group is listed in Table 1. Median surgery duration was 55 min, range: 25–226 min with no difference between the two groups (*p* = .605). A total of 27.2% of patients had an external ventricular drain (EVD) prior to primary shunt insertion, most commonly occurring in patients with tumor/malignancy, IVH, and ICH with or without IVH. A frontal drain was placed in 70.4% of patients. A vast majority received VP shunts (86.4%) followed by CP shunt (13.6%). None of the patients received atrial shunts. The most common type of valve inserted for the primary shunt insertion was the Medtronic Strata II valve (regular and small) with 68.8%, followed by the Medtronic PS Medical Ultra Small (non‐regulatory valve) with 16.3%. In five (6.2%) primary shunt insertions, ultrasound registration was used to place the ventricular drain. A consultant was the primary surgeon or first assist in 73 of 81 primary surgeries (90.1%), but no data in terms of years of practice or number of prior shunt surgeries performed by the individual surgeon were available.

Comparing the revision group with the non‐revision group, we found no differences in prior EVD, type of shunt (VP vs. CP), whether there were multiple passes of ventricular catheter or type of valve with the exception for Medtronic PS Medical Ultra Small (*p* = .006). Patients without frontal drain (i.e., occipital or cyst) were more likely to undergo revision (*p* = .045).

### 3.3 Revision surgery

A total of 125 revision surgeries were performed during the follow‐up period. 47 out of 81 (58.0%) patients underwent revision surgery at some point during follow‐up. A total of 42.0% of patients required revision surgery within the first year after shunt insertion. The number of shunt revisions ranged from 0 to 9, with a median of 1. An overview of the causes for revision surgery are presented in Table 2. The most common cause for the *first* revision was misplacement accounting for 29.8%, either of the proximal catheter or the distal catheter. Proximal occlusion/failure accounting for 25.5%, also including revisions with placement of additional ventricular drain(s), was the second most common cause for revision. Elective change from one valve to another, infection, and valve dysfunction were relatively uncommon causes of *first* revisions. The most common cause for *all* revisions regardless of whether it was a first or subsequent revision was proximal occlusion followed by misplacement. Infection was the cause of 9.6% of *all* revisions, and occurred in 5.8% of all procedures. In terms of all *procedures*, proximal occlusion was the most common cause for revision, followed by misplacement and infection.

**TABLE 2:** Revision surgery

	First revision	Later revisions	*p*‐value*	All revisions	All procedures
**Cause of revision**	*n* = 47	*n* = 78		*n* = 125 (%)	*n* = 206, %^†^
Misplacement	14 (29.8)	9 (11.5)	.011	23 (18.5)	11.2
Proximal occlusion	12 (25.5)	26 (33.3)	.358	38 (30.4)	18.4
Valve change	5 (10.6)	4 (5.1)	.295	9 (7.2)	4.4
Infection	3 (6.4)	9 (11.5)	.533	12 (9.6)	5.8
Valve dysfunction	3 (6.4)	3 (3.8)	.671	6 (4.8)	2.9
Length	2 (4.3)	0 (0.0)	.139	2 (1.6)	1.0
Exploration	2 (4.3)	5 (6.4)	.710	7 (5.6)	3.4
CSF leak	2 (4.3)	3 (3.8)	1.000	5 (4.0)	2.4
Shunt removal	2 (4.3)	1 (1.3)	.556	3 (2.4)	1.5
Distal	1 (2.1)	10 (12.8)	.051	11 (8.8)	5.3
Shunt conversion	1 (2.1)	1 (1.3)	1.000	2 (1.6)	1.0
Skin perforation	0 (0)	2 (2.6)	.527	2 (1.6)	1.0
New shunt/valve	0 (0)	5 (6.4)	.156	5 (4.0)	2.4

* *p*‐value expressed as difference between first and later revisions.

^†^ 81 procedures were primary procedures, and make up 39.3% of total 206 procedures.

Time from primary shunt insertion to first revision was median 18 weeks, range 0–425 weeks.

### 3.4 Shunt surgery complications

Overall complication rates in accordance with the grading system introduced by Ibanez et al. (2011) are presented in Table 3. A total of 30.6% of all procedures were followed by complications within 30 days. The most common complication was surgical grade IIb, accounting for approximately 18% of all complications, and 69.8% of all surgical complications. In approximately 5% of all procedures, medical complications occurred. Details of the complications which occurred within 30 days are given in Table 4.

**TABLE 3:** 30‐day overall complication rates

	Total	Medical	Surgical
	*n* = 206 (%)	*n* = 10 (%)	*n* = 53 (%)
**Grade I**			
Ia	2 (1.0)	1 (10.0)	1 (1.9)
Ib	13 (6.3)	7 (70.0)	6 (11.3)
**Grade II**			
IIa	1 (0.5)	0 (0.0)	1 (1.9)
IIb	37 (18.0)	0 (0.0)	37 (69.8)
**Grade III**			
IIIa	9 (4.4)	1 (10.0)	8 (15.1)
IIIb	0 (0.0)	0 (0.0)	0 (0.0)
**Grade IV**	1 (0.5)	1 (10.0)	0 (0.0)
**No complications**	143 (69.4)		

**TABLE 4:** Description of complications

Grade	Description	Total
Medical Ia	Epileptic seizure, not requiring AED	1
Surgical Ia	Track hematoma, not requiring surgery	1
Medical Ib	Pneumonia, treated w/ antibiotics	3
	UTI, treated w/ antibiotics	2
	Infection, unknown source, treated w/ antibiotics	2
Surgical Ib	Local wound infection, treated w/ antibiotics	4
	Epileptic seizure, requiring AED^a^	2
Surgical IIa	Dehiscent wound requiring closure	1
Surgical IIb	Shunt revision, any kind^b^	35
	Chronic subdural hematoma, requiring surgery twice	1
	CSF leakage, requiring duraplasty^c^	1
Medical IIIa	Respiratory failure, requiring reintubation	1
Surgical IIIa	Meningitis/CSF infection (also leading to shunt removal/externalization)	7
	N. recurrens paresis after traumatic intubation, and was reintubated	1
Medical IV	Death^d^	1

^a^ Both were cases where the underlying illness itself could cause epileptic seizures.

^b^ Revision surgery due to proximal failure, misplacement, CSF leak, adding an extra proximal catheter.

^c^ During primary shunt insertion, intraoperative bleeding from a bridging vein, requiring further opening of the dura, and consequently requiring revision surgery with duraplasty due to a postoperative CSF leak.

^d^ Patient with glioblastoma who clinically deteriorated with acute hydrocephalus and respiratory arrest; with a GCS of 3 and dilated pupils, the patient subsequently received an emergency shunt but died within 2 days due to herniation secondary to acute HC.

*Abbreviations*: AED, antiepileptic drugs; CSF, cerebrospinal fluid; UTI, urinary tract infection.

Binary univariable regression analysis showed age to be inversely associated with shunt revision *or* complication grade III *or* complication grade IV (*p* = .005, OR 0.87). Tumor/malignancy was inversely associated with shunt revision *or* grade III *or* grade IV complication (*p* = .015, OR 0.21), but failed to prove significant in a multivariable analysis. Gender and the most common etiologies (congenital HC, tumor/malignancy and arachnoid cyst) did not prove significantly associated with shunt revision *or* grade III *or* grade IV complication.

## 4 DISCUSSION

In this retrospective single center study, 81 children underwent primary shunt insertion in a 10‐year period. A total of 125 revisions were performed in this cohort during follow‐up. In total, 42% of patients required revision surgery within one year and nearly 60% required shunt revision during follow‐up. Hardware misplacement and proximal occlusion were the most common causes for revision, followed by infection. The revision group was significantly younger, and patients with congenital HC and MMC were more likely to experience revision surgery. Approximately one‐third experienced surgical or medical complications within 30‐days as classified by Ibanez et al (2011). This highlights that shunt complications are more than shunt failure or shunt infections. Measures to improve outcomes in the pediatric shunt population should be multi‐focused, and should target surgical precision in drain placement, perhaps especially in patients with higher risks of revisions. This could perhaps also improve long‐term patency. Lower revision rates will naturally also reduce other complication rates.

Despite shunt systems being in regular use for several decades, misplacements and proximal occlusions of the ventricular catheters still account for a great part of complication rates, as demonstrated in this study. Studies indicate that tools such as neuronavigation, ultrasound, and intraoperative imaging (fluoroscopy) improve accuracy, and in one study consequently reduced the rate of early return to the OR (AlAzri et al., 2017; Crowley et al., 2014; Janson et al., 2014). A review indicated level III evidence for the use of ultrasound and electromagnetic guidance for ventricular catheter placement (Flannery et al., 2014); at least one study was, however, most likely underpowered due to low number of participants. Studies that are clearly underpowered should not mistakenly be interpreted as lack of effect of said measure. In our study, neuronavigation or ultrasound guidance was only used in five patients during primary shunt insertion, but based on our findings, the routine use of neuronavigation or other tools to improve accuracy is probably the way to advance.

Postoperative scans were generally only performed in cases of clinical suspicion of shunt dysfunction. The number of revision rates due to misplacement may be an underestimation of actual misplacements. Although one prospective study failed to show improved shunt survival by using ultrasound or stereotactic guidance (Riva‐Cambrin et al., 2016), one cannot dismiss that improved placement, and avoiding the choroid plexus can reduce not only revision rates due to misplacement, but also perhaps due to occlusion. As the most frequently encountered revision causes; hence focus should be directed toward this.

In terms of infection rates already extensively examined in the literature, studies report greatly diverse infection rates, but generally ranging from 4% to 10% (Erps et al., 2018; Reddy et al., 2011; Riva‐Cambrin et al., 2016; Simon et al., 2014, 2009), but also higher (Bir et al., 2016; Simon et al., 2012). Our results are on the lower end. One study compared two cohorts almost 50 years apart, reporting few differences in shunt survival, and reporting 0.0% infection rate in the pediatric population, but the pediatric population was small (Mansson et al., 2017). Young age and perhaps more importantly, repeated revisions, have regularly been associated with increased risk of shunt infection (Arslan et al., 2018; Berry et al., 2008; Erps et al., 2018; Reddy et al., 2011; Simon et al., 2014, 2012). Some etiologies have also been associated with increased infection rates (Arslan et al., 2018; Tuli et al., 2000). Young age and etiology are undoubtedly intertwined; MMC has been suggested to be associated with infection (Arslan et al., 2018), but children with MMC usually undergo shunt insertion at a very young age, the latter probably being the most important factor. The use of antibiotic‐impregnated catheters (AIS) reduces infection rates (Mallucci et al., 2019; Sciubba et al., 2005). The regular use of an AIS should therefore be the gold standard. Only seven patients received AIS in the current study, but the number is too low to make any meaningful analysis on this. Although shunt infection is a serious complication, representing increased morbidity and mortality in shunt patients, it is not necessarily the most frequently encountered revision cause.

Revision surgery occurred most commonly within one year, findings similarly reported by others (Mansoor et al., 2020; Merkler et al., 2017; Stein & Guo, 2008). However, revision surgery also occurred after 8 years in the current study. One study followed patients for a minimum of 15 years and reported revision rate of 85%; 12.5% did not require their first revision before >10 years after initial shunt insertion, with the latest revision occurring as late as 17 years after shunt insertion (Stone et al., 2013). Other studies have similarly reported the need of revision surgery several decades after primary surgery (Bir et al., 2016; Paulsen et al., 2015; Vinchon et al., 2012). This indicates likely significant underreporting in a great portion of the current literature as few studies follow patients for such a long period of time. Significantly greater revision rates have been reported in studies with longer follow‐ups (Bir et al., 2016; Gupta et al., 2007; Stone et al., 2013). A shunt procedure in a pediatric patient indicates significant morbidity throughout the patient's lifetime.

Interestingly, 30% of patients experienced complications within 30 days. A surgical IIb complication was most commonly encountered, largely representing revision surgery, indicating high frequency of short shunt survival. We also detected other complications, such as extracranial infections, local wound infections, one instance of respiratory failure, and one recurrent laryngeal nerve paresis in relation to intubation. Similarly, we have found a high frequency of extracranial complications in an adult population (Mansoor et al., 2020). In all likelihood, extracranial complications represent significant morbidity with increased length of hospital stay. Focus on early mobilization, careful postoperative monitoring and wariness of extracranial complications seem to be important and manageable measures to consider. Reducing revision rates will also be a major step toward fewer complications in total.

The results of this study, particularly the high revisions rates, adds to already extensive literature where shunt complications represent a significant, but perhaps under‐communicated problem. There is a need for further research to improve treatment and reduce risk of complications in shunt patients. Authors have previously demonstrated immensely disproportioned cost/charges versus funding for HC in comparison to other conditions with similar incidence in the pediatric population (Simon et al., 2008).

In terms of etiologies presented in this study, the most noteworthy is perhaps the high proportion of patients with arachnoid cysts receiving shunts. Ten out of these 11 patients *did* undergo craniotomy with cyst fenestration prior to shunt insertion, but with inadequate results. There were relatively few patients with IVH compared to other studies; in four out of the five patients with ICH, imaging revealed blood in the ventricle system, but the primary diagnosis was not IVH, but rather parenchymal hemorrhage from arteriovenous malformations and other pathologies, which is why the two entities were not put together.

This study did not contemplate long‐term complications in terms of functional disabilities. Studies indicate that pediatric HC and shunt treatment are associated with cognitive challenges and reduced social functioning as well as mental health issues (Gupta et al., 2007; Kulkarni & Shams, 2007; Paulsen et al., 2015; Vinchon et al., 2012). Subsequent shunt complications, prolonged and frequent treatments for infections, and possibly also length of hospital stay (initial HC treatment) are factors that have been found to be associated with increased disability and reduced quality of life (Kulkarni & Shams, 2007); this also highlights the point in reducing the *overall* shunt complication rates, and not only one single factor.

Our study has several limitations, its retrospective design being one. Longer follow‐up would probably have indicated even higher frequency of shunt failure than currently reported. We suspect some overlap when it comes to etiologies, perhaps presenting difficulties when comparing the current results to other studies; authors have categorized HC differently in different studies, which may limit the generalizability of the results. Challenges of defining HC have previously been discussed, and due to the great variance in how authors have defined HC, it may affect the results in terms of specific etiologies being established risk factors for shunt failure.

## 5 CONCLUSIONS

We consider it worthwhile to reflect on all aspects that can reduce *overall* revision rates; whilst age and etiology are non‐modifiable factors frequently associated with the occurrence of revisions and complications, there should be a greater focus on any modifiable factors that can contribute to increased shunt survival and reduced morbidity. In the current study, misplacement and proximal occlusion accounted for the most frequent revisions. Increased attentiveness toward the early postoperative phase, with focus on prompt mobilization and prevention of extracranial infections, and so forth, are vital for improving care for these patients. All measures, however small, should be attempted to reduce *overall* complication rates.

## CONFLICT OF INTEREST

The authors declare no conflict of interest.

### PEER REVIEW

The peer review history for this article is available at https://publons.com/publon/10.1002/brb3.2390.

## Data Availability

Data available on request due to privacy/ethical restrictions.
